# Staged Surgical Treatment of Hidradenitis Suppurativa of the Axilla and Groin Using Lateral Thoracic and Anterolateral Thigh Flaps

**DOI:** 10.7759/cureus.89998

**Published:** 2025-08-13

**Authors:** Salim Al-lahham, Rami A Mesk, Bara Shraim, Ghanem Aljassem, Zaki T Alyazji

**Affiliations:** 1 Department of Plastic and Reconstructive Surgery, Hamad Medical Corporation, Doha, QAT; 2 College of Medicine and Health Sciences, Palestine Polytechnic University, Hebron, PSE

**Keywords:** anterolateral thigh (alt) flap, hidradenitis suppurativa (hs), hidradenitis suppurativa treatment, lateral thoracic flap, pedicle flap

## Abstract

Hidradenitis suppurativa (HS) is a chronic, relapsing inflammatory disease of the follicular epithelium that commonly affects apocrine gland-bearing areas such as the axillae, groin, and perineum. In advanced cases (Hurley Stage III), it is characterized by sinus tract formation, fibrosis, scarring, and recurrent abscesses. We present a case of a 20-year-old male patient with long-standing, treatment-resistant HS involving the bilateral axillae and groin. After failing multiple medical therapies over six years, he underwent staged surgical management with reconstruction using lateral thoracic fasciocutaneous flaps for axilla HS and with anterolateral thigh (ALT) perforator flaps for groin HS. Both procedures were successful with no flap loss or major complications, and the patient achieved full range of motion, excellent functional and cosmetic outcomes, and no recurrence at the six-month follow-up. This case highlights the effectiveness of wide excision with immediate regional flap reconstruction in advanced HS.

## Introduction

Hidradenitis suppurativa (HS) is a chronic, relapsing inflammatory skin disorder of the pilosebaceous-apocrine unit, primarily affecting intertriginous regions such as the axillae, groin, and perineum. Its pathogenesis is multifactorial, involving follicular occlusion, genetic predisposition, and dysregulated immune response [1]. Clinically, HS manifests as painful nodules, abscesses, sinus tracts, and scarring, with severity ranging from localized lesions to widespread tissue destruction. In severe cases, the disease leads to significant physical discomfort, psychological distress, social isolation, and a markedly diminished quality of life [2].

Medical therapies, including antibiotics, corticosteroids, and biologic agents, may provide temporary relief but are often insufficient in extensive, refractory disease. For Hurley stage II-III HS, especially with chronic sinus tract formation, surgical intervention becomes necessary. Techniques such as deroofing and wide local excision are endorsed by both the European S1 and North American guidelines [3,4].

Due to the inflammatory milieu and impaired wound healing, direct closure after excision is often associated with a high risk of dehiscence. Therefore, wide local excision to healthy margins followed by reconstruction using locoregional flaps is a more favorable strategy.

In this report, we present our preferred staged approach for managing extensive HS involving the axilla and groin, utilizing lateral chest flaps and anterolateral thigh (ALT) flaps for reconstruction, respectively.

## Case presentation

**Figure 1:**
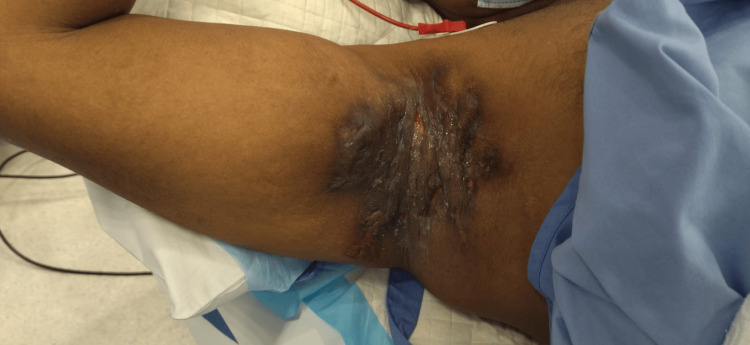
Preoperative image of the right axilla

**Figure 2:**
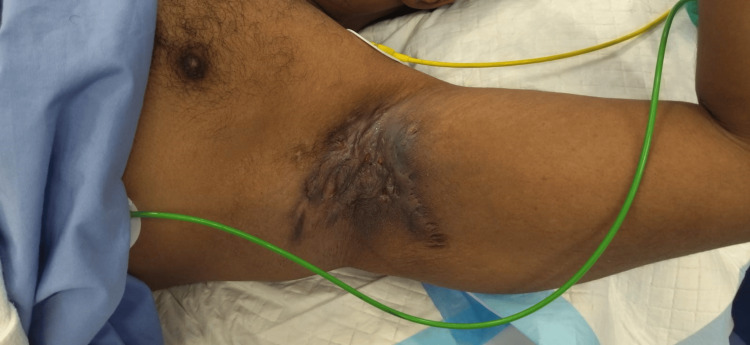
Preoperative image of the left axilla

**Figure 3:**
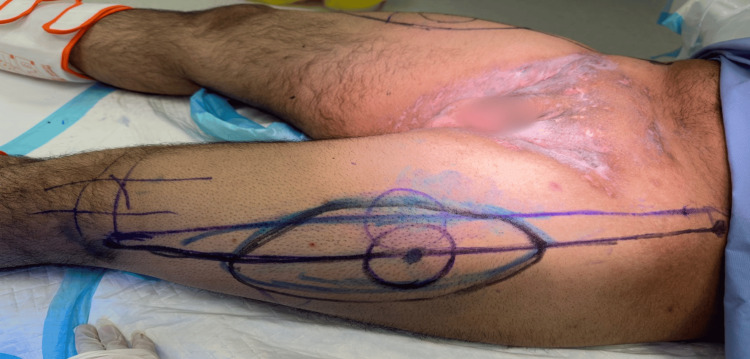
Preoperative image of the left groin and anterolateral thigh flap marking

**Figure 4:**
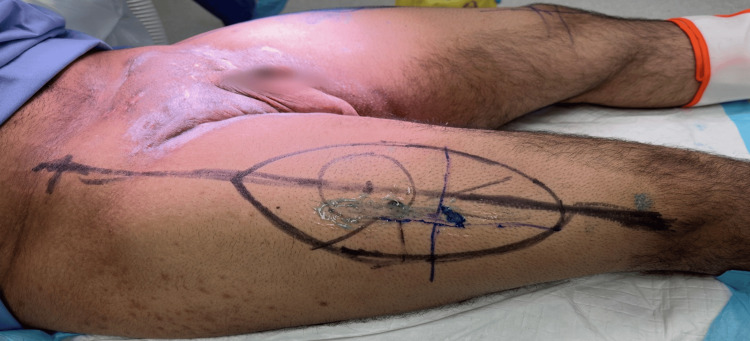
Preoperative image of the right groin and anterolateral thigh flap marking

The patient was a 20-year-old obese gentleman with no past medical history and a nonsmoker who presented to our clinic with severe HS affecting the axilla and groin. He was diagnosed six years ago with HS, Hurley stage 3, and referred from dermatology after no response to medical management, including immunotherapy (adalimumab 80 mg per week). On examination, there were multiple nodular lesions with discharge and sinus tracts involving both axilla and groin areas (Figures 1-4).

**Figure 5:**
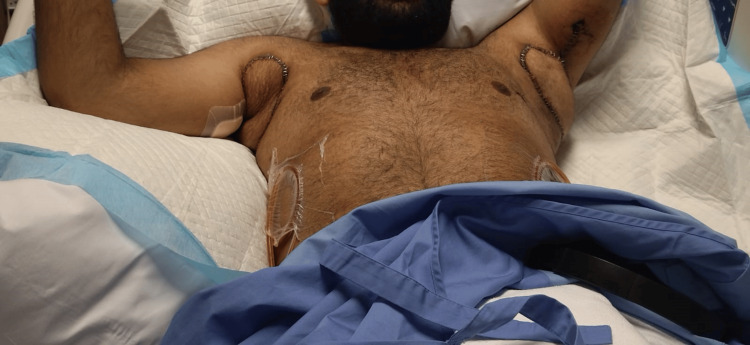
Postoperative image of the axilla

**Figure 6:**
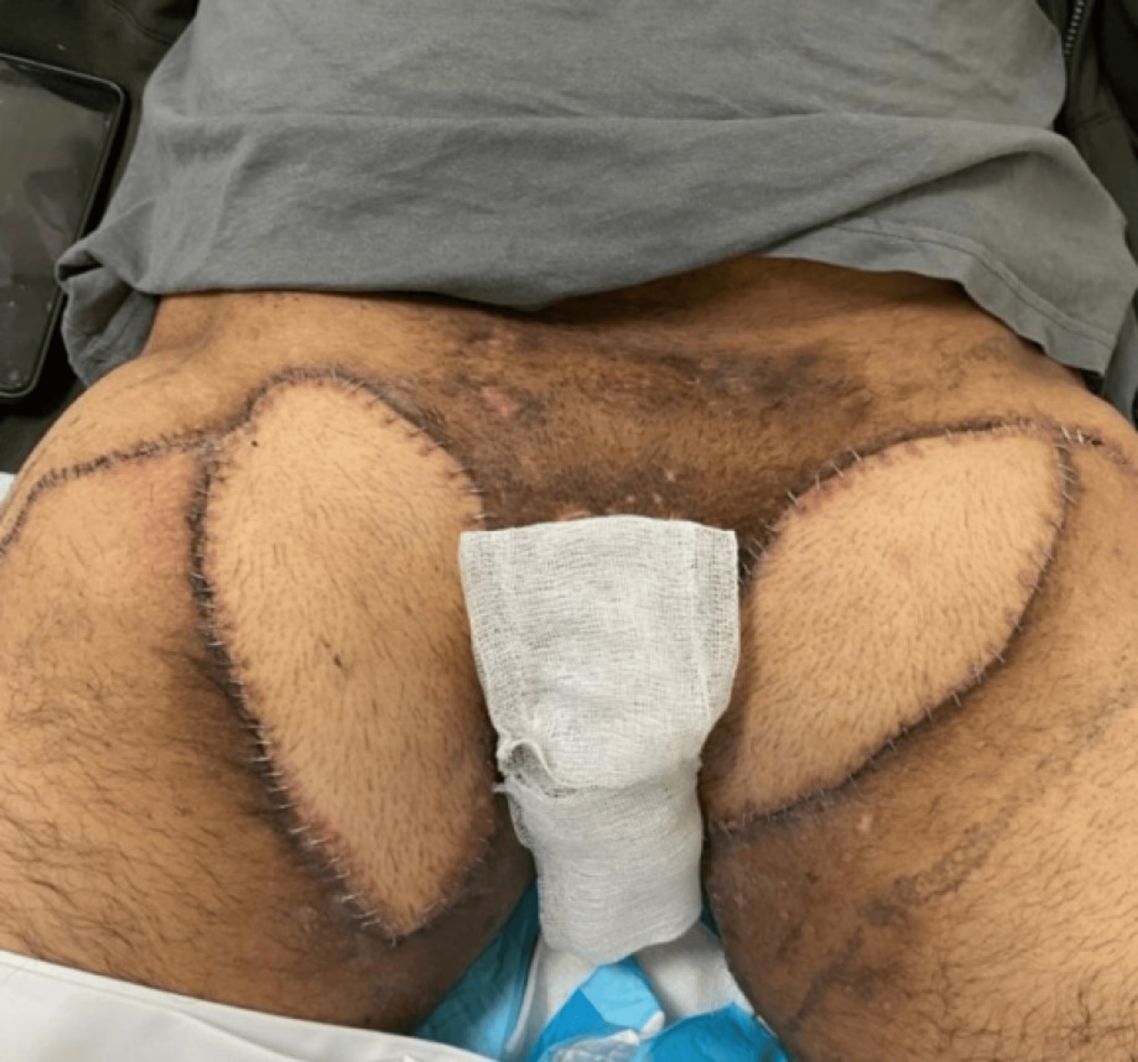
Postoperative image of the groin

We initially performed wide local excision of hidradenitis lesions in the axilla and reconstructed the defect with a lateral chest flap bilaterally under general anesthesia. The details of the flap design and elevation are detailed in the authors' previous work [5]. The course of treatment was uneventful, with full healing of the operative sites after three weeks of surgery (Figure 5). Subsequently, after around six months, we performed wide local excision of the groin lesions under spinal anesthesia. Reconstruction was achieved using pedicled anterolateral thigh perforator flaps (Figures 3, 4). After identifying the perforators on both sides using a handheld Doppler, the medial side of the flap was incised, and dissection of the pedicle was performed in a retrograde fashion, starting from the identified perforators and then tracing the descending branch of the lateral circumflex femoral artery; the maximum length of the pedicle was obtained. To deliver the flap to the defect, the pedicle was tunneled under the rectus femoris muscle to reduce kinking and gain extra length and freedom of motion for a tension-free inset. Flap perfusion was maintained and ensured before it was fixed and sutured in layers after insertion of J-VAC drains (Ethicon, Inc., Raritan, NJ). Finally, the donor sites were closed primarily in layers (Figure 6).

Our patient had a mild degree of wound dehiscence bilaterally, which was managed conservatively. He achieved full healing of the operative sites after four weeks. It is important to note that adalimumab was stopped four weeks before surgery and restarted after full healing of the operative sites. The patient was started on doxycycline one to two weeks before surgery and continued for a week after.

Lastly, the patient had scattered lesions in the suprapubic and scrotal areas as well as a lesion in the periphery of the right ALT flap, which was managed with excision and direct closure with no complications.

## Discussion

HS is a chronic and relapsing inflammatory skin condition originating from occlusion of the apocrine gland follicles of intertriginous areas. Extensive HS is often refractory to conservative treatments, and radical excision of the affected tissue is usually indicated.

Despite advances in the medical management of HS, surgical intervention is often required in severe cases to improve the aesthetic outcome of the affected area and decrease the recurrence rate. However, surgical treatment of HS is still challenging for many surgeons. The optimal surgical techniques in HS are variable, and there is no standard approach in the literature about which is the best surgical option for each of the most affected areas [6].

Reconstruction after wide local excision is critical not only for functional and cosmetic outcomes but also for minimizing recurrence. Several reconstructive options are available, each with its own advantages and limitations depending on the defect site, size, and patient factors.

For axillary HS, surgical reconstruction options include split-thickness skin grafts (STSGs), local advancement flaps such as Limberg or V-Y flaps, posterior arm flaps, and thoracodorsal artery perforator (TDAP) flaps. Skin grafts, while technically simple, have been associated with increased risk of contracture, delayed healing, poor durability, and less favorable cosmetic outcomes, particularly in intertriginous regions where mobility is high and moisture is constant [7,8]. Locoregional flaps, such as the lateral thoracic flap used in our case, provide a reliable fasciocutaneous option, preserving shoulder function, reducing the risk of axillary contracture, and minimizing donor site morbidity [9].

For groin HS, the reconstructive challenge is compounded by the anatomical complexity, movement, and proximity to urogenital structures. Closure by secondary intention or skin grafting may result in prolonged healing times, increased discomfort, and higher rates of recurrence or contracture. Myocutaneous flaps such as the gracilis flap have been used for groin defects, but they involve muscle sacrifice and may lead to functional impairment [10]. In contrast, the ALT flap, either pedicled or free, offers excellent pliability, consistent vascular anatomy, and a long pedicle length (~12-15 cm), which facilitates tension-free coverage of large or bilateral groin defects [11].

ALT flaps can be harvested as fasciocutaneous or myocutaneous, tailored based on defect characteristics. Compared to vertical rectus abdominis myocutaneous (VRAM) flaps or free perforator flaps like the deep inferior epigastric perforator (DIEP) or superior gluteal artery perforator (SGAP) flaps, the ALT flap avoids intra-abdominal harvest and microsurgical complexity while still providing significant bulk and surface area [12]. Moreover, in most patients, the ALT donor site can be closed primarily, reducing additional morbidity [13].

A staged surgical approach, as we present here, aligns with strategies recommended by several high-volume HS centers. It offers the advantages of allowing each region to heal before addressing the next, minimizing functional limitation from simultaneous bilateral reconstructions, and enabling better patient recovery and satisfaction [14].

In our experience, the lateral thoracic flap for axillary defects, followed by bilateral pedicled ALT flaps for groin reconstruction, offers a reliable and functional solution for extensive Hurley stage III disease. Although minor wound dehiscence occurred postoperatively, this is a common complication in HS surgeries due to the chronic inflammatory background and impaired tissue quality [15]. Nevertheless, our flap-based approach minimized severity and facilitated complete healing.

While our patient demonstrated satisfactory healing and no clinical recurrence at six months, it is important to recognize that hidradenitis suppurativa is a chronic condition, and recurrence may occur even years after surgical intervention. Several studies have reported variable long-term recurrence rates depending on the type of surgery, extent of excision, and patient risk factors.

For instance, Mehdizadeh et al. reported recurrence rates ranging from 13% to 70% depending on surgical technique and follow-up duration [6]. A systematic review by Bouazzi et al. found that wide excision followed by flap reconstruction had the lowest recurrence rate (6.7%) compared to grafts (27%) and secondary intention healing (22.7%) [16]. However, even with aggressive surgical management, long-term studies show recurrence can still occur two to five years postoperatively, particularly in patients with active smoking, obesity, or incomplete excision margins [17].

This case highlights how critical flap selection based on defect location, tissue characteristics, and patient context can significantly influence surgical outcomes. While no universal reconstruction algorithm exists for HS, growing literature supports the trend toward aggressive excision combined with immediate locoregional flap reconstruction in severe disease [4].

## Conclusions

This case highlights the effectiveness of wide local excision combined with immediate regional flap reconstruction in the management of advanced HS. Lateral thoracic flaps and ALT flaps are reliable options for axillary and groin reconstruction, respectively. A staged surgical approach provides a balanced strategy to achieve disease control, restore function, and improve long-term outcomes in patients with extensive HS unresponsive to medical therapy. Although our findings are encouraging, long-term follow-up and further studies involving larger patient cohorts are essential to substantiate the reproducibility and long-term success of this staged reconstructive strategy.
